# All hail reproducibility in microbiome research

**DOI:** 10.1186/2049-2618-2-8

**Published:** 2014-03-07

**Authors:** Jacques Ravel, K Eric Wommack

**Affiliations:** 1 Institute for Genome Sciences, University of Maryland School of Medicine, 801 W. Baltimore Street, Baltimore, MD 21201, USA; 2 Delaware Biotechnology Institute, University of Delaware, 15 Innovation Way, Newark, DE 19711, USA

## 

At its core, microbiome research relies on complex and sophisticated statistical analyses of large datasets and their associated metadata (e.g., experimental parameters, sample characteristics). Heavy reliance on big data has presented new challenges in communicating the details of complex analyses in a manner sufficient for others to replicate analytical workflows. Reproducibility is a pillar of sound research, and scientific journals need to embrace transparency and make every effort to enable reproducibility through comprehensive and clear reporting of analytical approaches. In this issue of *Microbiome*, a report by Meadow *et al.*[1] on the microbial communities of classroom surfaces sets a new bar for thoroughness in the availability of data, metadata, and analytical resources (code and scripts). It is our hope that this paper will serve as a template for the clever use of publicly available resources and code repositories to enable fully reproducible microbiome research.

“Scientific publications have at least two goals: (i) to announce a result and (ii) to convince readers that the result is correct… papers in experimental science should describe the results and provide a clear enough protocol to allow successful repetition and extension” [2]. Reproducibility and extension are only possible if: data is easily and freely accessible and delivered in format that adheres to international standards; and analysis workflows and scripts are embedded in the publication. Microbiome research is, by its nature, a multi-disciplinary endeavor where experimentalists often work with biostatisticians, mathematicians, computer scientists, or epidemiologists. At times, this multi-disciplinary character can result in a clash of scientific cultures with different approaches to openness, transparency and data release. For example, large sequence datasets and most importantly associated metadata have resulted from our work with epidemiologists [3,4]. However, the notion of releasing data and analysis scripts along with a publication has often been met with great surprise by our epidemiology colleagues. Now that microbiome research is transitioning from a descriptive and associative science to a translational science that will start impacting lives, we feel the time is right for the community to set standards for complete transparency and full reproducibility. Experimental science suffers each time there is a realization that a high profile report of a scientific finding is not reproducible. Over the long term, news stories of irreproducible science in the popular press can have lasting negative effects on the credibility of the scientific community in general [5]. Without reproducibility, microbiome science will battle to regain credibility and opportunities for scientific advancement will be lost.

Scientific journals should be at the forefront of efforts to ensure that data is accessible prior to publication and made available during the peer review process. Today, fortunately, there are numerous options for data release, such as among others, the NCBI Database of Genotypes and Phenotypes (dbGaP - http://www.ncbi.nlm.nih.gov/gap) and the Short Read Archive (SRA - http://www.ncbi.nlm.nih.gov/sra/), options selected by the Human Microbiome Project for example, or other services such as FigShare (http://www.figshare.com), which was used in the Meadow *et al* paper [1]. Data deposited into FigShare is permanently archived and redundantly backed up at major universities around the world through the CLOCKSS system (a not-for-profit venture started by libraries and publishers committed to ensuring long-term access to scholarly publications in digital format - http://www.clockss.org), and a permanent digital object identifier (DOI) is supplied with each dataset. Metadata associated with any dataset should also be made available, and in standard format with controlled ontology. Standards such as the minimum information about a marker gene sequence (MIMARKS) or the minimum information about any (x) sequence (MIxS) [6] are community driven standards that if fully adopted would enhance the long-term scientific use of microbiome datasets.

Data availability is critical but detailed descriptions of the procedures used in the processing of raw data and statistical analyses are equally important for reproducibility. Simply providing scripts and workflow is not enough; data and code have to be understandable to be reproducible. Hence, commenting and versioning is essential and should be included in the publication of scripts. There are several tools available depending on the statistical package or programing language. For example, iPython notebook (http://www.ipython.org/ipython-doc/dev/interactive/notebook.html) for python scripts enables commenting and tutorials for documenting use cases. Popular tools such as DigiNorm developed by Dr. C. Titus Brown (Michigan State University) use iPython notebook (http://www.ged.msu.edu/papers/2012-diginorm/) and it is no mistake that the best documented tools often turn out to be more frequently used by microbiome researchers. Statistical analyses in microbiome research increasingly rely on the R statistical language [7]. The R Markdown language simplifies creation of fully-reproducible statistical analysis [8], and has been implemented in packages such as Sweave [9] or knitr [10]. Combined with GitHub (http://www.github.com), a code versioning repository, scripts can be run and analytical outcomes from reported datasets can be fully reproduced. Dozens of other packages are available for commenting and release of workflow and scripts. Again, Meadow and co-authors [1] used both knitr and GitHub in making their statistical workflow and code publicly available. We applaud the efforts of initiatives such as the Minimum Information About a Bioinformatics investigation (MIABi) [11], which seeks to advance standards for bioinformatics activities that will improve the persistence, reproducibility, and disambiguation of code. Ultimately, these practices will improve transparency and reproducibility. Moving forward *Microbiome* will seek to raise the bar for reproducibility in microbiome research by asking authors to provide easy access to data and code that will ultimately enrich our vibrant and growing research field.

## Competing interests

The authors declare that they have no competing interests.

## Authors’ contribution

JR and KEW wrote the manuscript. Both authors read and approved the final manuscript.
